# An ecological view on the correlates of sedentary behavior in Brazilian adolescents: a cross-sectional study with network analysis

**DOI:** 10.1186/s44167-024-00052-w

**Published:** 2024-05-23

**Authors:** José Ywgne, Leonardo G. O. Luz, Mabliny Thuany, Cayo Lima, Raphael Araujo, Ellen Silva, Kleberton Magalhães, Paulo Bandeira, Danilo R. Silva

**Affiliations:** 1 Federal University of Sergipe, Postgraduate Program in Physical Education, Av. Marechal Rondon, s/n, Jardim Rosa Elze, São Cristóvão, SE CEP 49100-000 Brazil; 2 Faculty of Health Sciences, Universidad Autónoma de Chile, Providencia, Chile; 3 Laboratory of Kineanthropometry, Physical Activity and Health Promotion, Federal University of Alagoas, Arapiraca, AL Brazil; 4 Faculty of Sports, University of Porto, Porto, Portugal; 5 Graduation Program in Health Sciences, Londrina State University, Londrina, Brazil; 6 Regional University of Cariri, Crato, CE Brazil; 7 The Research Center for Sport and Physical Activity (CIDAF), University of Coimbra, Coimbra, Portugal

**Keywords:** Complex systems, Adolescent, Sedentary lifestyle, Brazil

## Abstract

**Background:**

Sedentary behavior has been identified as a public health concern. The present study analyzed the correlates of sedentary behavior in Brazilian adolescents through network analysis, in the light of an ecological approach.

**Methods:**

The sample consisted of 126,354 adolescents, participating in the fourth edition of the Brazilian National School Health Survey. The variables were grouped into domains, namely intrapersonal, interpersonal, perceived environment, school variables, food variables, active behavior domains, and leisure sedentary behavior. The data were analyzed using network analysis.

**Results:**

The results reinforce that sedentary behavior is independent of the assessed active behavior domains, despite being weakly related to physical activity outside physical education classes (− 0.073). The variable with the greatest *closeness* and one of the variables with the greatest *expected influence* in the model was having internet at home, which was associated with private schools (− 0.230), having a cell phone (0.378), having a computer or notebook at home (0.409), and a greater weekly frequency of watching television (0.169). Furthermore, having internet at home was strongly associated with an increase in sedentary behavior (− 0.197), which, in turn, was linked to greater consumption of treats (0.067) and sodas (0.052).

**Conclusions:**

We concluded that having internet at home is associated with longer sedentary time, which, in turn, is associated with greater consumption of foods of poorer nutritional quality. Interventions on sedentary behavior should be specific for population subgroups and consider actions in different levels.

**Supplementary Information:**

The online version contains supplementary material available at 10.1186/s44167-024-00052-w.

## Background

Sedentary behavior can be defined as a set of activities with energy expenditure ≤ 1.5 metabolic equivalents, in sitting, lying, or reclining positions, during the waking period [1]. Time in sedentary behavior increases throughout life [2] and is associated with the development of chronic non-communicable diseases, such as hypertension, diabetes, and obesity [3]. Globally, high levels of sedentary behavior are reported in adolescents [4]. In Brazil, three in ten adolescents spend more than 2 h in leisure-time sedentary behavior per day [5].

The sedentary behavior of adolescents is determined by a set of factors ˗ sociodemographic characteristics, school characteristics, use of electronic devices, socioeconomic level, environment, internet access, and poor diet [6–8]. Therefore, understanding the correlates (factors associated with sedentary behavior) and determinants (those with a causal relationship) of sedentary behavior through an ecological perspective enables assertive actions to be proposed to change this problem [9, 10]. In this context, the ecological theory of Sallis et al. [11], adapted by Owen et al. [12] for sedentary behavior, highlights the need to consider the interrelationships between correlates of different domains, such as intrapersonal and interpersonal characteristics, the built environment, and the political environment.

Population-based studies verified the relationship between sedentary behavior and the level of physical activity [13], sociodemographic variables (sex, maternal education, family economic level) [14], and behavioral variables (consumption of ultra-processed foods) [15]. However, complex problems are difficult to understand and resolve due to the relationships between their multiple dynamic causes, thus requiring the use of statistical approaches that consider the complexity of this system [16].

Network analysis provides an overall structural organization, or topology, of the phenomenon and the roles played by specific variables in the network, which can thus be analyzed in a way that other approaches cannot offer [17]. Previous studies on the correlates of sedentary behavior [13, 15, 18] have used traditional linear analyses, such as regression analysis. Although this type of analysis contributes to understanding of correlates, this approach is not suitable for examining a complex system of interconnected variables, since it does not aid visualization of variables that can influence sedentary behavior in a broad and complex way. Therefore, conducting a comprehensive analysis of variables is essential. This process aids in identifying emerging patterns and observing key variables that possess the potential to significantly reshape the system [19]. In this way, essential information is generated for the development of intervention programs [20] aimed at reducing sedentary behavior.

In this sense, the objective of the current study is to verify, through social network analysis, the associations between variables of intrapersonal and interpersonal characteristics, perceived environment, school variables, food variables, domains of active behavior, and leisure sedentary behavior in Brazilian adolescents.

## Methods

### Sample and study design

This is a cross-sectional study, with data from the Brazilian National School Health Survey (PeNSE), 2019 edition. The PeNSE aims to identify risk and protective factors for the health of adolescents and all editions of the survey were carried out by the Brazilian Institute of Geography and Statistics (IBGE), in partnership with the Ministry of Health.

The PeNSE 2019 carried out a single sample, capable of estimating the indicators of interest with greater geographic detail. Furthermore, the survey was designed to provide results representative of students aged 13 to 18 or over, enrolled between the 7th year of elementary school and the 3rd year of high school with regular attendance in public and private schools throughout Brazil [21]. The PeNSE 2019 was designed to estimate population parameters (proportions or prevalence) in different geographic domains: each of the 26 capitals of the Federation Units and the Federal District, in addition to 26 non-capital cities in each state [21]. The sample in the current study included 126,354 adolescents, of both sexes, aged between 13 and 18 years or older. The sampling process can be found in previous publications [21]. The collection period took place between 04/08/2019 and 09/30/2019. The investigation was approved by the National Research Ethics Committee nº 3,249,268 [21]. Furthermore, we followed the STROBE *checklist* [22]. The *checklist* can be found in supplementary Table 1.

Data collection was carried out using a smartphone on which the structured questionnaires were downloaded. The data were collected through two questionnaires; the student questionnaire, which was completed by students in the selected class, and the school questionnaire, which was completed by the director or person in charge of the selected school.

Based on the information provided by the instrument, for the present study, the variables were chosen based on the ecological theory for sedentary behavior [12]. The variables were divided into the following domains: intrapersonal, interpersonal, perceived environment, domains of sedentary behavior, and context of behavior (access, characteristics, and political environment). However, in the present study, the political environment was not considered, given the lack of information. Furthermore, food, school, and active behavior variables (domains of physical activity) were added, as we saw the need to more broadly encompass possible variables that could be related to sedentary behavior.

The categorization of each variable, as well as the respective encodings used for network analysis are presented in supplementary Table 2.

### Intrapersonal variables

Sex (male and female), age (less than 13 years of age, 13 to 15 years of age, 16 or 17 years of age, and 18 years or more), maternal education (did not study, incomplete primary education, completed primary education, incomplete secondary education, complete secondary education, incomplete higher education, complete higher education), how you consider your body (very thin, thin, normal, fat, very fat), and self-perception of health (very good, good, regular, bad, very bad) were considered.

### Interpersonal variable

The number of friends (no friends, one to two friends, three or more friends) was used as the main variable at the interpersonal level.

### Perceived environment

The environment was assessed through the perception of safety on the home-school route, in which participants answered the following question: “In the last 30 days, on how many days did you not go to school because you didn’t feel safe at school?” (no day, one day, two days, three days, four days, or five days or more). We classified “feeling unsafe” based on the report of having not gone to school on at least one day.

### School variables

School variables were: the school variables were administrative dependency (public or private) and type of municipality (capital/non-capital).

### Feeding variables

For the food variables (ate beans, ate fruit, ate treats, and drank soda), considering the seven days immediately prior to the survey, the possible answers were: (a) I didn't eat/didn't drink it; (b) 1 day; (c) 2 days; (d) 3 days; (e) 4 days; (f) 5 days; (g) 6 days; (h) every day. These food variables were then recategorized for descriptive analyses: (a) no days; (b) 1–3 days; (c) 4–6 days; (d) every day.

### Active behavior

The domains of physical activity were organized into active commuting (average daily time accumulated by the student, traveling from home to school and from school to home, on foot or by bicycle), physical activity in physical education class (average time accumulated, in the seven days prior to the survey, that the student performed physical activity or sport during physical education classes at school), and physical activity outside of physical education classes (average daily time accumulated by the student practicing an extracurricular physical activity), always considering the seven days prior to the date of the research, and reported in minutes [21]. The students were asked how many days they performed physical activity and how long the respective activity lasted [21]. For descriptive analysis, the domains were dichotomized into: “yes” for those who reported practicing at least one day of physical activity in the week prior to the survey and “no” for those who reported not practicing PA on any day in the previous week. For network analysis, minutes (continuous variable) were computed in each PA domain.

### Sedentary behavior

Sedentary leisure behavior was used, based on the following question: “On a typical weekday, how long do you spend sitting, watching television, using the computer, playing video games, talking to friends, or performing other activities while seated?” (do not count Saturday, Sunday, holidays, and time spent sitting at school). The possible answers were: (a) up to 1 h per day; (b) more than 1 h and up to 2 h per day; (c) more than 2 h and up to 3 h per day; (d) more than 3 h and up to 4 h per day; (e) more than 4 h and up to 5 h per day; (f) more than 5 h and up to 6 h per day; (g) more than 6 h and up to 7 h per day; (h) more than 7 h and up to 8 h per day; (i) more than 8 h a day; (j) not informed. To include only valid data, students who did not report time spent in sedentary behavior were excluded from the sample. The variable was then recategorized for descriptive analysis (≤ 2 h; > 2 h and ≤ 4 h; > 4 h and ≤ 6; or > 6 h) [18].

### Contexts of behavior: access and characteristics

Behavioral contexts: access and characteristics were used as variables, possession of a cell phone, internet at home, and a computer were assessed using a dichotomous indication (yes/no). The frequency of eating while watching television or using the cell phone (every day of the week, 5 to 6 days a week, 3 to 4 days a week, 1 to 2 days a week, I don't usually eat while doing anything else) was used as an indicator of aggregation of unhealthy habits.

### Data analysis

The characterization of the sample was carried out considering the intrapersonal and interpersonal characteristics, built environment, school variables, dietary variables, active behavior, sedentary behavior, and behavioral context (access and characteristics) domains. The information presented prevalence (%) and 95% confidence intervals (CI95%), for the total sample without missing data and with the original PeNSE 2019 sample.

Network analysis serves as our primary analytical method, offering a comprehensive means to discern intricate relationship patterns and network structures. Through topology, which represents the visual framework of connections, and the weight matrix, which quantifies correlations, we can uncover pivotal characteristics within the network. Therefore, network analysis was used to analyze the complex relationship between variables from the intrapersonal, interpersonal, built environment, school variables, nutrition variables, active behavior, sedentary behavior, and behavior context (access and characteristics) domains. The “Fruchterman-Reingold” algorithm was applied [23]. The data were shown in relative space in which variables are grouped by approximation [17]. The “Markov pair random fields” algorithm was used to improve the accuracy of the network. The algorithm adds an “L1” penalty (regularized neighborhood regression). The regulation is estimated by a less complete selection and contraction operator (Lasso) that controls the sparse network [24]. The extended Bayesian information criterion (EBIC) for selecting the regularization parameter Lambda was observed. EBIC uses a hyperparameter (y) that determines how much EBIC selects sparse models. The y value was determined to be 0.25 (range 0 to 0.25).

Network analysis uses regularized least absolute shrinkage and selection operator (LASSO) algorithms to obtain the precision matrix (weight matrix). The weight matrix is a correlation matrix that quantifies the network topology by representing each variable as a node and each correlation as an edge [25]. By varying the width of the edges according to the magnitude of the correlation, the structure of the correlation matrix can be visualized. Just like a partial correlation, the values range from − 1 to 1. However, network analysis differs in the way the statistical procedure is performed. We use a 'least absolute contraction and selection operator' (LASSO) [26] applied to the estimation of partial correlation networks. LASSO performs well in estimating partial correlation networks [27], and this results in reducing some small weak edge estimates to exactly zero, resulting in a sparse network [28]. As the elements of the network weight matrix more closely reflect the dependency structure of the relationships between variables, the weight matrix becomes more accurate and defensible [29]. When standardized, this matrix represents the associations between variables in the network. The network is presented in a graph that includes variables (nodes) and relationships (lines). Blue lines represent positive associations and red lines represent negative associations. The thickness and intensity of the lines represent the magnitude of the associations. The weight matrix quantifies the associations, taking into account the correlations between the variables.

Another measure is centrality, *Closeness* (a group of nodes highly interconnected with each other and poorly connected with nodes outside this group) and *Expected Influence* (a variable with greater sensitivity in the network, thus having greater power to change the network) [17]. Furthermore, network analysis considers sampling weight, weights were assigned to network links based on the frequency or relative importance of interactions between nodes. Additionally, centrality measures and other network statistics were calculated taking these sampling weights into account, ensuring that the results adequately reflected the structure of the source population. Analyses were performed using JASP software (version 16.1).

## Results

The total sample of valid students from PeNSE 2019 is made up of 159,245 students, however it is important to highlight that depending on the variable, the total sample of valid data may be different. However, in the present study 32,891 were excluded due to missing data or because they did not provide information about their mother's education. Supplementary Table 3 shows the amount of missing data removed for each study variable, as well as the order of exclusion. Therefore, the current article included a sample of 126,354 adolescents (65,784 girls). Table 1 (full table can be seen in supplementary Table 4 presents descriptive data from the total sample of the present study and the original PeNSE sample. It is observed more missing data in specific groups, such as boys, younger adolescents among those who have less access to cellphone, computer and internet at home, as shown in Table 1.

**Table 1:** Characteristics of the domains intrapersonal, perceived environment, school variables, active behavior, sedentary behavior, and the contexts of behavior (access and characteristics) for the sample in the present study (n = 126,354) and PeNSE total sample

Domains	Variable	Present study			PeNSE total sample		
		Total	%	95% CI	Total	%	95% CI
Intrapersonal	Sex						
	Boys	60,570	47.9	47.7–48.2	78,011	49.1	49.0–49.4
	Girls	65,784	52.1	51.8–52.3	80,788	50.9	50.6–51.1
	Age						
	< 13 years	18,202	14.4	14.2–14.6	25,642	16.1	16.0–16.3
	13–15 years	65,025	51.5	51.2–51.7	82,389	51.9	51.6–52.1
	16–17 years	36,373	28.8	28.5–29.0	42,509	25.8	26.5–27.0
	≥ 18 years	6,754	5.3	5.2–5.5	8,276	5.2	5.1–5.3
Perceived environment	Perception of insecurity						
	Yes	12,040	9.5	9.4–9.7	15,717	10.0	9.8–10.1
	No	114,314	90.5	90.3–90.6	142,033	90.0	89.7–90.0
School	Administrative dependence						
	Public	60,076	47.6	47.3–47.8	81,496	51.2	50.9–51.4
	Private	66,278	52.4	52.2–52.7	77,749	48.8	48.6–49.1
	Type of Municipality						
	Capital	65,374	51.7	51.5–52.0	81,906	51.4	51.2–51.7
	Non-capital	60,980	48.3	48.0–48.5	77,339	48.6	48.3–48.8
Active behavior	Active commuting PA						
	No	62,066	49.1	48.8–49.4	75,763	47.8	47.6–48.1
	Yes	64,288	50.9	50.6–51.1	82,620	52.2	51.9–52.4
	Physical education classes PA						
	No	50,897	40.3	40.0–40.5	63,927	40.4	40.1–40.6
	Yes	75,457	59.7	59.4–60.0	94,456	59.6	59.4–60.0
	Extra-physical education classes PA						
	No	41,787	33.1	32.8–33.3	53,424	33.7	34.1–34.5
	Yes	84,567	66.9	66.7–67.2	105,158	66.3	66.1–66.5
Sedentary behavior	Leisure						
	Up to 2 h	36,492	28.9	28.6–29.1	47,928	30.5	30.3–30.8
	> 2 h and < 4 h	36,288	28.7	28.5–29.0	43,543	27.8	27.5–28.0
	> 4 h and < 6 h	26,250	20.8	20.5–21.0	31,336	19.9	20.0–20.2
	> 6 h	27,324	21.6	21.4–21.8	34,168	21.8	21.6–22.0
Access and characteristics	Have cell phone						
	Yes	111,256	88.1	87.9–88.2	138,065	86.7	86.6–86.9
	No	15,098	11.9	11.8–12.1	21,121	13.3	13.1–13.4
	Have computer						
	Yes	89,035	70.5	70.1–70.7	107,223	67.3	67.1–67.6
	No	37,319	29.5	29.3–29.8	51,944	32.6	32.4–32.9
	Internet access at home						
	Yes	116,130	91.9	91.7–92.1	144,380	90.7	90.5–90.8
	No	10,224	8.1	7.9–8.2	14,796	9.3	9.1–9.4

CI: confidence interval; For the domains of physical activity, Active commuting PA, Physical education classes PA, Extra-physical education classes PA, "yes" are those who reported practicing at least 1 min of physical activity in the week prior to the survey and "no" are those who reported not practicing PA for any minutes in the week prior to the survey; Present study: Sample after removing all missing items for any study variable; PeNSE’s total sample: Sample with original valid data for each variable

It is observed that 51.5% (95% CI 51.2–51.7) of students were aged 13 to 15. Regarding the education level of the students' mothers, 39.1% (95% CI 38.8–39.4) completed higher education. It is also noteworthy that 91.9% (95% CI 91.7–92.1) of students have access to the internet at home. In addition, 88.1% (95% CI 87.9–88.2) have a cell phone and (95% CI 70.1–70.7) have a desktop or laptop at home. Regarding time spent in sedentary behavior, it was observed that 71.1% of students spent more than 2 h a day in leisure-time sedentary behavior.

**Fig. 1:**
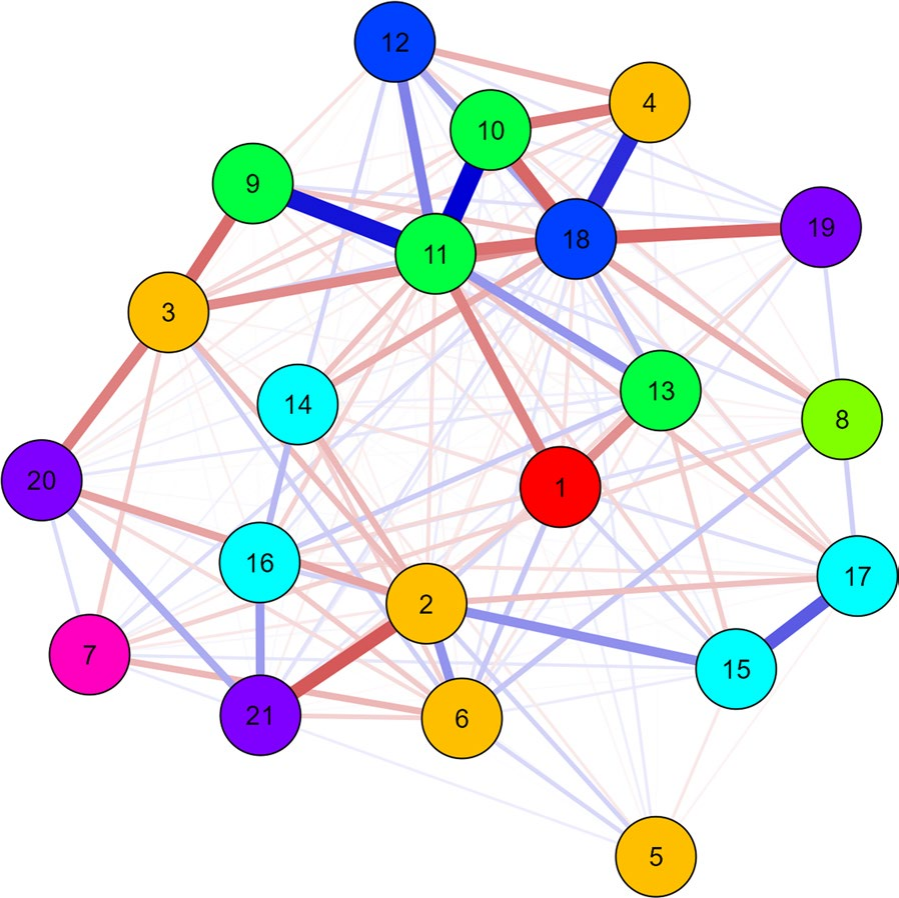
Network topology for the relationship between domains, intrapersonal, interpersonal, perceived environment, school variables, nutrition variables, active behavior, sedentary behavior, and behavior context (access and characteristics). Blue edge: positive association; Red edge: negative association. Red node: leisurely sedentary behavior; Yellow node: intrapersonal; Pink node: interpersonal; Dark green node: perceived environment; Dark blue node: school variables; Light blue node: nutrition variables; Lilac node: active behavior; Light green node: contexts of behavior. 1—Sedentary behavior; 2—Sex; 3—Age; 4—Maternal education; 5—How do you consider your body; 6—Self-perception of health; 7- Number of friends; 8—Perception of insecurity; 9—Has cell phone; 10—Has a computer or laptop; 11—Has internet at home; 12—Type of Municipality; 13—Frequency of eating while watching television; 14—Bean consumption; 15—Treat consumption; 16—Fruit consumption; 17—Soda intake; 18—Administrative dependency 19—Active commuting physical activity; 20—Physical education classes physical activity; 21—Extra-physical education classes physical activity. The sign of the results of the relationships (positive or negative) will depend on how the variable was categorized. The categorization can be seen in Supplementary Table 2

The main results of the network analysis (Fig. 1 and Table 2) indicated that girls are more sedentary than boys (− 0.033). Furthermore, sedentary behavior and physical activity outside of physical education classes had a negative association (− 0.073), that is, students who showed greater sedentary behavior had less time practicing physical activity outside of education classes. The variable with the greatest *closeness* and one of the variables with the greatest *expected influence* in the model is having internet at home, which is associated with private schools (− 0.230), having a cell phone (0.378), having a computer or notebook at home (0.409), and a greater weekly frequency of watching television (0.169). Furthermore, having internet at home has a negative association (− 0.197) with sedentary behavior, which, in turn, is linked to greater consumption of treats (0.067) and sodas (0.052). In addition, more time spent in sedentary behavior was associated with worse body perception (0.025), as well as worse self-perception of health (0.091).

**Table 2:**
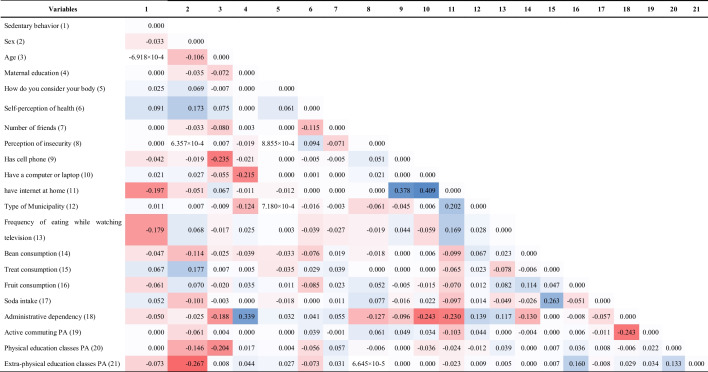
Association between the study variables from the perspective of network analysis

PA: Physical activity. The sign of the results of the relationships (positive or negative) will depend on how the variable was categorized. The categorization can be seen in Supplementary Table 2

In relation to food, students who consumed more fruit reported more time in physical activity outside physical education class (0.160).

Table 3 presents the results of the centrality indicators. The variables consumption of treats (1.675), fruits (1.203), the type of municipality (1.092), and having internet at home (0.935) presented the highest *expected influence* values. In relation to *closeness* (variables that presented a greater grouping of nodes highly interconnected with each other), the variables internet access (1.631), administrative dependence (1.483), and age (1.250) demonstrated higher values.

**Table 3:** Measures of centrality by study variable

Variable	Closeness	Expected influence
Sedentary behavior	0.334	− 1.072
Sex	0.639	− 1.016
Age	1.250	− 2.395
Maternal education	0.119	− 0.006
How do you consider your body	− 2.710	0.586
Self-perception of health	− 0.187	0.621
Number of friends	− 1.597	− 0.099
Perception of insecurity	− 0.394	0.333
Have cell phone	1.060	0.295
Have a computer or laptop	0.650	− 0.036
Have internet at home	1.631	0.935
Type of Municipality	− 0.452	1.092
Frequency of eating while watching television	0.042	0.611
Bean consumption	0.139	− 0.981
Treat consumption	− 0.371	1.675
Fruit consumption	− 0.714	1.203
Soda intake	− 0.492	0.227
Administrative dependency	1.483	− 1.786
Active commuting PA	− 0.290	− 0.202
Physical education classes PA	0.250	− 0.313
Extra-physical education classes PA	− 0.390	0.329

PA: Physical activity

## Discussion

This study verified the multilevel correlates of sedentary behavior in Brazilian adolescents. The main findings indicate that adolescents with more time in sedentary behavior were related to less time in physical activity outside of physical education classes. The variable with the greatest closeness and one of the variables with the greatest expected *influence* in the model is having internet at home, which is associated with students who are enrolled in private schools, have a cell phone, have a computer or notebook at home, and have a higher weekly frequency of eats by watching television or using the cell phone. Furthermore, adolescents who have internet at home tend to spend more time in sedentary behavior, which in turn is linked to greater consumption of treats and sodas. Thus, increasingly widespread internet access appears to play a central role in triggering sedentary behaviors, poor diet, and reduced time spent in leisure-time physical activity.

Non-modifiable intrapersonal variables, such as sex and age were associated with sedentary behavior [14], with girls having longer exposure time to sedentary behavior than boys [14] and older individuals having longer exposure time to sedentary behaviors in relation to younger people [14]. Other correlates have been associated with excessive time in sedentary behavior, such as mother's higher level of education [30] and a greater number of friends [9]. Our findings corroborate the literature regarding the relationship with non-modifiable variables (sex and age), however we did not find a direct association with the mother's education and number of friends. These differences may be related to the type of analysis used, since when we observe the complexity that exists between all the variables in a single system, we can see in the network topology both the mother's education level and the number of friends, linked to other variables, and following a “path” until reaching sedentary behavior. We emphasize that this type of analysis advances the literature, bringing results to complex problems.

We observed that more sedentary students report less time in physical activities outside of physical education classes [31]. In addition to the possible direct relationship between the behaviors, these results may suggest that economic issues could interfere with both behaviors [32]. We can observe that students who practice more physical activity outside of physical education classes, practice more physical activity in physical education classes, consequently, they are students whose mothers have more education, which, in turn means that they have more access to electronic devices, that had a direct link with greater time in leisure-time sedentary behavior.

The context of sedentary behaviors has also been studied [14, 18, 30]. Having access to a cell phone, internet, eating meals while watching television, consuming treats, and drinking sodas were associated with greater time in sedentary leisure behavior [14, 18]. The findings of the present study corroborate previous studies; however, we report advances in the way of observing the interconnection between the variables. For example, it is possible to observe from the network topology that the variable with the greatest expected influence (the most sensitive variable in the network, therefore the one with the greatest power to change relationships), the consumption of treats is directly associated, not only with sedentary behavior, but also intrapersonal (self-perception of body and self-perception of health) and interpersonal (number of friends) variables. Furthermore, it is highlighted that a worse self-perception of body and health is directly linked to a longer period of sedentary behavior, that is, this evidence suggests that the approach to changing behaviors in young people should be focused on multiple behaviors rather than specific interventions, considering that several aspects can influence these behaviors.

In general, the differences observed between our findings and the literature may be related to methodological aspects, such as the statistical procedures performed. Network analysis allows the investigation of complex patterns, taking into account the non-linearity of the relationships present between the variables of a system [17]. In this sense, we consider the correlates of sedentary behavior as a complex system, which emerges through a multifactorial interaction. The structural organization of network topology, phenomena, and the roles played by specific variables in the network can be analyzed in a way that other statistical approaches cannot provide [17]. The literature lacks additional studies that take into account the complexity that exists in the correlates of sedentary behavior. It is also necessary to carry out longitudinal studies to observe prospective relationships and intervention studies that consider the complexity of interactions between variables to reduce sedentary behavior.

Some limitations of the current work should be recognized. The question regarding sedentary behavior makes it impossible to distinguish between behaviors that occur within this group. It is also noteworthy that physical activity and sedentary behavior were self-reported. On the other hand, questionnaires have been used in other studies carried out in the context of physical activity and sedentary behavior [30], presenting good reliability among adolescents [33]. The main positive points include the originality of the statistical procedures and theoretical conception used. We utilized a representative sample of Brazilian adolescents, although some groups may lack representativeness due to missing data. Nevertheless, this survey stands as the most comprehensive examination of Brazilian adolescents to date.

We can therefore highlight that the findings of this study carry some practical implications. Public policies should be developed with the aim of educating the population, especially children and adolescents, about the problems of excessive internet use. While clear cutoffs for classifying excessive internet use remain elusive, the undeniable trend of increasing internet usage underscores the importance of promoting awareness about the associated risks, particularly the implications of prolonged online time. Moreover, our identification of treat consumption as the variable exerting the greatest influence on the system suggests a correlation with unhealthy habits. Consequently, health interventions should address sedentary behavior and dietary habits collectively as primary components of school policies.

## Conclusions

We concluded that when considering the complexity of the correlates of sedentary behavior, intrapersonal variables, such as sex and access to internet and electronic equipment, as well as poor diet, should be highlighted. It is also noteworthy that students who practice more physical activities outside of physical education classes tend to present less sedentary behavior.

## Supplementary Information

Supplementary Material 1.

## Data Availability

The datasets generated and/or analysed during the current study are available in Edições | IBGE.
