# Supplementary material for: An ecological view on the correlates of sedentary behavior in Brazilian adolescents: a cross-sectional study with network analysis
Source: J Act Sedentary Sleep Behav. 2024 May 23;3:13. doi: 10.1186/s44167-024-00052-w (PMC11960373; doi:10.1186/s44167-024-00052-w)
Supplement: Supplementary file 1 — Supplementary Material 1. [file 44167_2024_52_MOESM1_ESM.docx]

**Supplementary material**

**Supplementary Table 1.** STROBE Statement checklist of items that should be included in reports of observational studies.

|  | Item No | | Recommendation | Done? |
| --- | --- | --- | --- | --- |
| **Title and abstract** | 1 | | (*a*) Indicate the study’s design with a commonly used term in the title or the abstract | Page 1 |
|  |  |  | (*b*) Provide in the abstract an informative and balanced summary of what was done and what was found | Page 1 |
| Introduction | | | |  |
| Background/rationale | 2 | | Explain the scientific background and rationale for the investigation being reported | Page 2 |
| Objectives | 3 | | State specific objectives, including any prespecified hypotheses | Page 3 |
| Methods | | | |  |
| Study design | 4 | | Present key elements of study design early in the paper | Page 4 |
| Setting | 5 | | Describe the setting, locations, and relevant dates, including periods of recruitment, exposure, follow-up, and data collection | Page 4 |
| Participants | 6 | | (a) Give the eligibility criteria, and the sources and methods of selection of participants | Page 4 |
|  |  |  |  |  |
| Variables | 7 | | Clearly define all outcomes, exposures, predictors, potential confounders, and effect modifiers. Give diagnostic criteria, if applicable | Page 5-8 |
| Data sources/ measurement | 8* | | For each variable of interest, give sources of data and details of methods of assessment (measurement). Describe comparability of assessment methods if there is more than one group | Page *5-8* |
| Bias | 9 | | Describe any efforts to address potential sources of bias | Not applicable |
| Study size | 10 | | Explain how the study size was arrived at | Not applicable |
| Quantitative variables | 11 | | Explain how quantitative variables were handled in the analyses. If applicable, describe which groupings were chosen and why | Page 7-8 |
| Statistical methods | 12 | | (*a*) Describe all statistical methods, including those used to control for confounding | Page 7-8 |
|  |  |  | (*b*) Describe any methods used to examine subgroups and interactions | Page 7-8 |
|  |  |  | (*c*) Explain how missing data were addressed | Page 8 |
|  |  |  | (*d*) If applicable, describe analytical methods taking account of sampling strategy | Not applicable |
|  |  |  | (*e*) Describe any sensitivity analyses | Not applicable |
|  |  | |  |  |
| Results |  | | |  |
| Participants | 13* | (a) Report numbers of individuals at each stage of study—eg numbers potentially eligible, examined for eligibility, confirmed eligible, included in the study, completing follow-up, and analysed  (b) Give reasons for non-participation at each stage  (c) Consider use of a flow diagram | | Page 9 |
|  |  |  |  | Page 9 |
|  |  |  |  | Not applicable |
| Descriptive data | 14* | (a) Give characteristics of study participants (eg demographic, clinical, social) and information on exposures and potential confounders | | Page 9-10 |
|  |  | (b) Indicate number of participants with missing data for each variable of interest | | Page 9 |
|  |  |  | |  |
| Outcome data | 15* | *Cross-sectional study—*Report numbers of outcome events or summary measures | | Page 10 |
|  |  |  |  |  |
|  |  |  |  |  |
| Main results | 16 | (*a*) Give unadjusted estimates and, if applicable, confounder-adjusted estimates and their precision (eg, 95% confidence interval). Make clear which confounders were adjusted for and why they were included | | Page 9 |
|  |  | (*b*) Report category boundaries when continuous variables were categorized | | Supplementary |
|  |  | (*c*) If relevant, consider translating estimates of relative risk into absolute risk for a meaningful time period | | Not applicable |
| Other analyses | 17 | Report other analyses done—eg analyses of subgroups and interactions, and sensitivity analyses | | Page 9-10 |
| Discussion |  | | |  |
| Key results | 18 | Summarise key results with reference to study objectives | | Page 11 |
| Limitations | 19 | Discuss limitations of the study, taking into account sources of potential bias or imprecision. Discuss both direction and magnitude of any potential bias | | Page 14 |
| Interpretation | 20 | Give a cautious overall interpretation of results considering objectives, limitations, multiplicity of analyses, results from similar studies, and other relevant evidence | | Page 11 - 14 |
| Generalisability | 21 | Discuss the generalisability (external validity) of the study results | | Page 13-14 |
| Other information |  | | |  |
| Funding | 22 | Give the source of funding and the role of the funders for the present study and, if applicable, for the original study on which the present article is based | | Not applicable |

*Give information separately for cases and controls in case-control studies and, if applicable, for exposed and unexposed groups in cohort and cross-sectional studies.

**Note:** An Explanation and Elaboration article discusses each checklist item and gives methodological background and published examples of transparent reporting. The STROBE checklist is best used in conjunction with this article (freely available on the Web sites of PLoS Medicine at http://www.plosmedicine.org/, Annals of Internal Medicine at http://www.annals.org/, and Epidemiology at http://www.epidem.com/). Information on the STROBE Initiative is available at [www.strobe-statement.org](http://www.strobe-statement.org);

**Supplementary Table 2.** The categorization of each variable, as well as their respective coding that were used for network analysis.

| Domains | Variables | Categorization | Coding of the variables used in network analysis |
| --- | --- | --- | --- |
| Intrapersonal | Sex | Boys | 1 |
|  |  | Girls | 2 |
|  | Age | Under 13 years old | 1 |
|  |  | 13 to 15 years | 2 |
|  |  | 16 to 17 years | 3 |
|  |  | 18 years or more | 4 |
|  | What level of education (degree) did your MOTHER study or study? | Did not study | 1 |
|  |  | Incomplete primary education | 2 |
|  |  | Complete primary education | 3 |
|  |  | Incomplete secondary education | 4 |
|  |  | Complete secondary education | 5 |
|  |  | Incomplete higher education | 6 |
|  |  | Complete higher education | 7 |
|  | How do you consider your body? | Very thin | 1 |
|  |  | Thin | 2 |
|  |  | Normal | 3 |
|  |  | Fat | 4 |
|  |  | Very fat | 5 |
|  | Self-perception of health | Very good | 1 |
|  |  | Good | 2 |
|  |  | Regular | 3 |
|  |  | Bad | 4 |
|  |  | Very bad | 5 |
| Interpersonal | How many close friends do you have? | No friends | 1 |
|  |  | 1 – 2 friends | 2 |
|  |  | 3 or more friends | 3 |
|  |  | No friends | 4 |
| Perceived environment | IN THE LAST 30 DAYS, how many days did you not go to school because you didn't feel safe AT SCHOOL? | 1 day | 1 |
|  |  | 2 days | 2 |
|  |  | 3 days | 3 |
|  |  | 4 days | 4 |
|  |  | 5 days or more | 5 |
| School | Administrative dependence | Public | 1 |
|  |  | Private | 2 |
|  | Type of municipality | Capital | 1 |
|  |  | Non-capital | 2 |
| Food consumption | IN THE LAST 7 DAYS, how many days have you eaten beans? | I haven't eaten beans in the last 7 days | 1 |
|  |  | 1 day | 2 |
|  |  | 2 days | 3 |
|  |  | 3 days | 4 |
|  |  | 4 days | 5 |
|  |  | 5 days | 6 |
|  |  | 6 days | 7 |
|  |  | Every day | 8 |
|  | IN THE LAST 7 DAYS, how many days have you eaten fresh fruit or fruit salad? | I haven't eaten any fresh fruit or fruit salad in the last 7 days | 1 |
|  |  | 1 day | 2 |
|  |  | 2 days | 3 |
|  |  | 3 days | 4 |
|  |  | 4 days | 5 |
|  |  | 5 days | 6 |
|  |  | 6 days | 7 |
|  |  | Every day | 8 |
|  | IN THE LAST 7 DAYS, how many days have you eaten sweet treats, such as candies, confectionery, chocolates, chewing gum, bonbons, lollipops, and others? | I haven't eaten any treats in the last 7 days | 1 |
|  |  | 1 day | 2 |
|  |  | 2 days | 3 |
|  |  | 3 days | 4 |
|  |  | 4 days | 5 |
|  |  | 5 days | 6 |
|  |  | 6 days | 7 |
|  |  | Every day | 8 |
|  | IN THE LAST 7 DAYS, how many days have you had soda? | I haven't had soda in the last 7 days | 1 |
|  |  | 1 day | 2 |
|  |  | 2 days | 3 |
|  |  | 3 days | 4 |
|  |  | 4 days | 5 |
|  |  | 5 days | 6 |
|  |  | 6 days | 7 |
|  |  | Every day | 8 |
| Active behavior | Active commuting | In minutes | Continuous |
|  | Physical education classes PA | In minutes | Continuous |
|  | Extra-physical education classes PA | In minutes | Continuous |
| Leisure sedentary behavior | How many hours a day do you usually sit, watch television, play video games, use a computer, cell phone, tablet or do other activities while sitting? (DO NOT count Saturday, Sunday, holidays or sitting time at school) | Up to 1 hour per day | 1 |
|  |  | More than 1 hour up to 2 hours per day | 2 |
|  |  | More than 2 hours up to 3 hours a day | 3 |
|  |  | More than 3 hours up to 4 hours a day | 4 |
|  |  | More than 4 hours up to 5 hours a day | 5 |
|  |  | More than 5 hours up to 6 hours a day | 6 |
|  |  | More than 6 hours up to 7 hours a day | 7 |
|  |  | More than 7 hours up to 8 hours a day | 8 |
|  |  | More than 8 hours a day | 9 |
| Access and characteristics | Do you have a cell phone? | Yes | 1 |
|  |  | No | 2 |
|  | Do you have a computer or laptop in your home? | Yes | 1 |
|  |  | No | 2 |
|  | Do you have internet access in your home? | Yes | 1 |
|  |  | No | 2 |
|  | At your meals, how often do you eat while doing something else (watching TV, using your computer or cell phone)? | Every day of the week | 1 |
|  |  | 5 to 6 days a week | 2 |
|  |  | 3 to 4 days a week | 3 |
|  |  | 1 to 2 days a week | 4 |
|  |  | I don't usually eat while doing something else | 5 |

Note: For the domains of physical activity, Active commuting PA, Physical education classes PA, Extra-physical education classes PA, "yes" are those who reported practicing at least one minute of physical activity in the week prior to the survey and "no" are those who reported not practicing PA for any minutes in the week prior to the survey.

| **Variable** | **Missing** | **Missing excluded** | **Deletion order by variable** |
| --- | --- | --- | --- |
| Type of Municipality | 0 | 0 | 1º |
| Administrative dependency | 0 | 0 | 2º |
| Sex | 446 | 446 | 3º |
| Age | 429 | 410 | 4º |
| Have cell phone | 59 | 59 | 5º |
| Have a computer or laptop | 78 | 45 | 6º |
| Have internet at home | 69 | 29 | 7º |
| Maternal Education | 27991 | 27778 | 8º |
| Frequency of eating while watching television | 202 | 95 | 9º |
| Bean consumption | 244 | 103 | 10º |
| Treat consumption | 299 | 117 | 11º |
| Fruit consumption | 298 | 92 | 12º |
| Soda consumption | 233 | 72 | 13º |
| Sedentary behavior | 2270 | 1550 | 14º |
| Number of friends | 534 | 214 | 15º |
| Perception of insecurity | 1495 | 649 | 16º |
| Self-perception of health | 1255 | 252 | 17º |
| How do you consider your body | 1489 | 203 | 18º |
| Active commuting PA | 862 | 325 | 19º |
| Physical education classes PA | 865 | 312 | 20º |
| Extra-physical education classes PA | 663 | 140 | 21º |

**Supplementary table 3.** Missing from the original PeNSE database and final study sample (126,354) after excluding missing ones.

PA: Physical Activity.

**Supplementary Table 4.** Characteristics of the intrapersonal domains, perceived environment, school variables, active behavior, sedentary behavior and behavioral contexts (access and characteristics) for the sample in the present study (n = 126,354) and PeNSE’s total sample.

| Domains | Variable | Present study | | |  | PeNSE total sample | | |
| --- | --- | --- | --- | --- | --- | --- | --- | --- |
|  |  | Total | % | 95%CI |  | Total | % | 95%CI |
|  | **Sex** |  |  |  |  |  |  |  |
|  | Boys | 60,570 | 47.9 | 47.7-48.2 |  | 78,011 | 49.1 | 49.0-49.4 |
|  | Girls | 65,784 | 52.1 | 51.8-52.3 |  | 80,788 | 50.9 | 50.6-51.1 |
|  |  |  |  |  |  |  |  |  |
|  | **Age** |  |  |  |  |  |  |  |
|  | <13 years | 18,202 | 14.4 | 14.2-14.6 |  | 25,642 | 16.1 | 16.0-16.3 |
|  | 13-15 years | 65,025 | 51.5 | 51.2-51.7 |  | 82,389 | 51.9 | 51.6-52.1 |
|  | 16-17 years | 36,373 | 28.8 | 28.5-29.0 |  | 42,509 | 25.8 | 26.5-27.0 |
|  | ≥18 years | 6,754 | 5.3 | 5.2-5.5 |  | 8,276 | 5.2 | 5.1-5.3 |
|  |  |  |  |  |  |  |  |  |
|  | **Maternal education** |  |  |  |  |  |  |  |
|  | Did not study | 4,131 | 3.3 | 3.2-3.4 |  | 4,541 | 3.5 | 3.4-3.6 |
|  | Incomplete primary education | 17,617 | 13.9 | 13.7-14.1 |  | 18,589 | 14.2 | 14.0-14.3 |
|  | Complete primary education | 6,898 | 5.5 | 5.3-5.6 |  | 7,230 | 5.5 | 5.4-5.6 |
|  | Incomplete secondary education | 9,080 | 7.2 | 7.0-7.3 |  | 9,484 | 7.2 | 7.1-7.4 |
|  | Complete secondary education | 28,183 | 22.3 | 22.1-22.5 |  | 29,175 | 22.2 | 22.0-22.4 |
| Intrapersonal | Incomplete higher education | 11,056 | 8.8 | 8.6-8.9 |  | 11,425 | 8.7 | 8.5-8.9 |
|  | Complete higher education | 49,389 | 39.1 | 38.8-39.4 |  | 50,810 | 38.7 | 38.4-40.0 |
|  |  |  |  |  |  |  |  |  |
|  | **How do you consider your body?** |  |  |  |  |  |  |  |
|  | Very thin | 8,087 | 6.4 | 6.3-6.5 |  | 10,429 | 6.6 | 6.5-6.7 |
|  | Thin | 27,126 | 21.5 | 21.2-21.7 |  | 33,528 | 21.3 | 21.0-21.5 |
|  | Normal | 62,062 | 49.1 | 48.8-49.4 |  | 78,686 | 49.9 | 49.6-50.1 |
|  | Fat | 25,148 | 19.9 | 19.7-20.1 |  | 30,285 | 19.2 | 19.0-19.4 |
|  | Very fat | 3,931 | 3.1 | 3.0-3.2 |  | 4,828 | 3.1 | 3.0-3.1 |
|  |  |  |  |  |  |  |  |  |
|  | **Self-perception of health** |  |  |  |  |  |  |  |
|  | Very good | 37,946 | 30.0 | 29.8-30.3 |  | 49,069 | 31.1 | 30.8-31.3 |
|  | Good | 48,488 | 38.4 | 38.1-38.6 |  | 59,815 | 37.9 | 37.6-38.1 |
|  | Regular | 32,876 | 26.0 | 25.8-26.3 |  | 40,435 | 25.6 | 25.4-25.8 |
|  | Bad | 5,430 | 4.3 | 4.2-4.4 |  | 6,579 | 4.2 | 4.1-4.3 |
|  | Very bad | 1,614 | 1.3 | 1.2-1.3 |  | 2,092 | 1.3 | 1.3-1.4 |
|  |  |  |  |  |  |  |  |  |
| Interpersonal | **Number of friends** |  |  |  |  |  |  |  |
|  | No friends | 3,936 | 3.1 | 3.0-3.2 |  | 5,240 | 3.3 | 3.2-3.4 |
|  | 1 – 2 friends | 23,579 | 18.6 | 18.4-18.9 |  | 28,977 | 18.3 | 18.1-18.4 |
|  | 3 or more friends | 98,839 | 78.2 | 78.0-78.4 |  | 124,494 | 78.4 | 78.2-78.6 |
|  |  |  |  |  |  |  |  |  |
| Perceived environment | **Perception of feeling unsafe** |  |  |  |  |  |  |  |
|  | Yes | 12,040 | 9.5 | 9.4-9.7 |  | 15,717 | 10.0 | 9.8-10.1 |
|  | No | 114,314 | 90.5 | 90.3-90.6 |  | 142,033 | 90.0 | 89.7-90.0 |
|  |  |  |  |  |  |  |  |  |
| School | **Administrative dependence** |  |  |  |  |  |  |  |
|  | Public | 60,076 | 47.6 | 47.3-47.8 |  | 81,496 | 51.2 | 50.9-51.4 |
|  | Private | 66,278 | 52.4 | 52.2-52.7 |  | 77,749 | 48.8 | 48.6-49.1 |
|  |  |  |  |  |  |  |  |  |
|  | **Type of Municipality** |  |  |  |  |  |  |  |
|  | Capital | 65,374 | 51.7 | 51.5-52.0 |  | 81,906 | 51.4 | 51.2-51.7 |
|  | Non-capital | 60,980 | 48.3 | 48.0-48.5 |  | 77,339 | 48.6 | 48.3-48.8 |
|  |  |  |  |  |  |  |  |  |
| Food | **Beans** |  |  |  |  |  |  |  |
|  | No days | 16,031 | 12.7 | 12.5-12.9 |  | 20,420 | 12.8 | 12.7-13.0 |
|  | 1-3 days | 32,284 | 25.6 | 25.3-25.8 |  | 39,907 | 25.1 | 24.9-25.3 |
|  | 4-6 days | 33,277 | 26.4 | 26.1-26.6 |  | 39,560 | 24.9 | 24.7-25.1 |
|  | Every day | 44,762 | 35.4 | 35.2-35.7 |  | 59,114 | 37.2 | 36.9-37.4 |
|  |  |  |  |  |  |  |  |  |
|  | **Fruit** |  |  |  |  |  |  |  |
|  | No days | 31,970 | 25.3 | 25.1-25.5 |  | 42,122 | 26.5 | 26.3-26.7 |
|  | 1-3 days | 45,387 | 35.9 | 35.7-36.2 |  | 56,889 | 35.8 | 35.6-36.0 |
|  | 4-6 days | 26,309 | 20.8 | 20.6-21.0 |  | 31,731 | 20.0 | 19.8-20.2 |
|  | Every day | 22,688 | 18.0 | 17.7-18.2 |  | 28,205 | 17.7 | 17.6-17.9 |
|  |  |  |  |  |  |  |  |  |
|  | **Treats** |  |  |  |  |  |  |  |
|  | No days | 14,377 | 11.4 | 11.2-11.5 |  | 19,626 | 12.3 | 12.9-12.5 |
|  | 1-3 days | 56,680 | 44.8 | 44.6-45.1 |  | 71,353 | 44.9 | 44.6-45.1 |
|  | 4-6 days | 31,554 | 25.0 | 24.7-25.2 |  | 37,647 | 23.7 | 23.5-23.9 |
|  | Every day | 23,743 | 18.8 | 18.6-19.0 |  | 30,320 | 19.1 | 18.9-19.3 |
|  |  |  |  |  |  |  |  |  |
|  | **Sodas** |  |  |  |  |  |  |  |
|  | No days | 33,757 | 26.7 | 36.5-27.0 |  | 42,707 | 26.9 | 26.6-27.1 |
|  | 1-3 days | 62,161 | 49.3 | 48.9-49.5 |  | 78,088 | 49.1 | 48.9-49.3 |
|  | 4-6 days | 20,932 | 16.6 | 16.4-16.8 |  | 25,742 | 16.2 | 16.0-16.4 |
|  | Every day | 9,504 | 7.5 | 7.4-7.7 |  | 12,475 | 7.8 | 7.7-8.0 |
|  |  |  |  |  |  |  |  |  |
| Active behavior | **Active commuting PA** |  |  |  |  |  |  |  |
|  | No | 62,066 | 49.1 | 48.8-49.4 |  | 75,763 | 47.8 | 47.6-48.1 |
|  | Yes | 64,288 | 50.9 | 50.6-51.1 |  | 82,620 | 52.2 | 51.9-52.4 |
|  |  |  |  |  |  |  |  |  |
|  | **Physical education classes PA** |  |  |  |  |  |  |  |
|  | No | 50,897 | 40.3 | 40.0-40.5 |  | 63,927 | 40.4 | 40.1-40.6 |
|  | Yes | 75,457 | 59.7 | 59.4-60.0 |  | 94,456 | 59.6 | 59.4-60.0 |
|  |  |  |  |  |  |  |  |  |
|  | **Extra-physical education classes PA** |  |  |  |  |  |  |  |
|  | No | 41,787 | 33.1 | 32.8-33.3 |  | 53,424 | 33.7 | 34.1-34.5 |
|  | Yes | 84,567 | 66.9 | 66.7-67.2 |  | 105,158 | 66.3 | 66.1-66.5 |
|  |  |  |  |  |  |  |  |  |
| Sedentary behavior | **Leisure** |  |  |  |  |  |  |  |
|  | Up to 2h | 36,492 | 28.9 | 28.6-29.1 |  | 47,928 | 30.5 | 30.3-30.8 |
|  | >2h and < 4h | 36,288 | 28.7 | 28.5-29.0 |  | 43,543 | 27.8 | 27.5-28.0 |
|  | > 4h and <6h | 26,250 | 20.8 | 20.5-21.0 |  | 31,336 | 19.9 | 20.0-20.2 |
|  | >6h | 27,324 | 21.6 | 21.4-21.8 |  | 34,168 | 21.8 | 21.6-22.0 |
|  |  |  |  |  |  |  |  |  |
|  | **Have cell phone** |  |  |  |  |  |  |  |
|  | Yes | 111,256 | 88.1 | 87.9-88.2 |  | 138,065 | 86.7 | 86.6-86.9 |
|  | No | 15,098 | 11.9 | 11.8-12.1 |  | 21,121 | 13.3 | 13.1-13.4 |
|  |  |  |  |  |  |  |  |  |
|  | **Have computer** |  |  |  |  |  |  |  |
| Access and characteristics | Yes | 89,035 | 70.5 | 70.1-70.7 |  | 107,223 | 67.3 | 67.1-67.6 |
|  | No | 37,319 | 29.5 | 29.3-29.8 |  | 51,944 | 32.6 | 32.4-32.9 |
|  |  |  |  |  |  |  |  |  |
|  | **Internet access at home** |  |  |  |  |  |  |  |
|  | Yes | 116,130 | 91.9 | 91.7-92.1 |  | 144,380 | 90.7 | 90.5-90.8 |
|  | No | 10,224 | 8.1 | 7.9-8.2 |  | 14,796 | 9.3 | 9.1-9.4 |
|  |  |  |  |  |  |  |  |  |
|  | **Frequency of eating while watching television** |  |  |  |  |  |  |  |
|  | Every day of the week | 57,070 | 45.2 | 44.9-45.4 |  | 73,697 | 46.3 | 46.1-46.5 |
|  | 5 to 6 days a week | 10,267 | 8.1 | 8.0-8.3 |  | 11,923 | 7.4 | 7.4-7.6 |
|  | 3 to 4 days a week | 12,029 | 9.5 | 9.4-9.7 |  | 13,990 | 8.8 | 8.6-8.9 |
|  | 1 to 2 days a week | 12,610 | 10.0 | 9.8-10.1 |  | 15,196 | 9.6 | 9.4-9.7 |
|  | I don't usually eat while doing something else | 34,378 | 27.2 | 27.0-27.4 |  | 44,337 | 27.9 | 27.6-28.1 |

CI: confidence interval; For the domains of physical activity, Active commuting PA, Physical education classes PA, Extra-physical education classes PA, "yes" are those who reported practicing at least one minute of physical activity in the week prior to the survey and "no" are those who reported not practicing PA for any minutes in the week prior to the survey; Present study: Sample after removing all missing items for any study variable; PeNSE’s total sample: Sample with original valid data for each variable.
